# Study on X-ray Induced Two-Dimensional Thermal Shock Waves in Carbon/Phenolic

**DOI:** 10.3390/ma14133553

**Published:** 2021-06-25

**Authors:** Dengwang Wang, Yong Gao, Sheng Wang, Jie Wang, Haipeng Li

**Affiliations:** Department of Nuclear Science and Technology, Xi’an Jiaotong University, Xi’an 710000, China; wdw21s@stu.xjtu.edu.cn (D.W.); gaoyong1108@stu.xjtu.edu.cn (Y.G.); wangjie1@xjtu.edu.cn (J.W.); lihaipeng@xjtu.edu.cn (H.L.)

**Keywords:** thermal shock wave, Carbon/Phenolic (C/P), constitutive relation, intense pulse X-ray, energy deposition

## Abstract

Carbon/Phenolic (C/P), a typical anisotropic material, is an important component of aerospace and often used to protect the thermodynamic effects of strong X-ray radiation. In this paper, we establish the anisotropic elastic-plastic constitutive model, which is embedded in the in-house code “RAMA” to simulate a two-dimensional thermal shock wave induced by X-ray. Then, we compare the numerical simulation results with the thermal shock wave stress generated by the same strong current electron beam via experiment to verify the correctness of the numerical simulation. Subsequently, we discuss and analyze the rules of thermal shock wave propagation in C/P material by further numerical simulation. The results reveal that the thermal shock wave represents different shapes and mechanisms by the radiation of 1 keV and 3 keV X-rays. The vaporization recoil phenomenon appears as a compression wave under 1 keV X-ray irradiation, and X-ray penetration is caused by thermal deformation under 3 keV X-ray irradiation. The thermal shock wave propagation exhibits two-dimensional characteristics, the energy deposition of 1 keV and 3 keV both decays exponentially, the energy deposition of 1 keV-peak soft X-ray is high, and the deposition depth is shallow, while the energy deposition of 3 keV-peak hard X-ray is low, and the deposition depth is deep. RAMA can successfully realize two-dimensional orthotropic elastoplastic constitutive relation, the corresponding program was designed and checked, and the calculation results for inspection are consistent with the theory. This study has great significance in the evaluation of anisotropic material protection under the radiation of intense X-rays.

## 1. Introduction

Carbon/Phenolic (C/P), as an advanced type of anisotropic composite material, and has been widely used in the field of aerospace [1,2]. This material has the advantages of low density, high strength, oxidation resistance, low coefficient of thermal expansion and mechanical stability at high temperature [3]. Due to the complex external environment of aviation and aerospace, such materials may be facing high-speed collision, radiation, and other dynamic loading environments [4,5]. Under intense pulsed X-ray irradiation, a large amount of energy is rapidly deposited on the surface of the material and decays rapidly inside, which causes a large temperature and pressure gradient in the material. On the other hand, the irradiated material undergoes adiabatic expansion due to a rapid increase in specific internal energy, so with large radiation that in turn has a recoil effect on the material, the material on the light-facing surface would vaporize and then be ejected. The combined effects of these factors will form unsteady stress waves (i.e., X-ray thermal shock waves) within the material [6,7]. When thermal shock waves propagate to the interface or the free surface with low impact impedance, they would unload and form sparse waves that propagate in the opposite direction. They interact with the sparse section of the incident thermal shock waves to produce tensile stress, which would crack the material or delaminate the delamination interface and thus damage the material [8]. With the wide application of C/P composite in spacecraft and missile hulls, the prediction of dynamic response and internal damage of structures caused by explosion, impact or shock wave under the action of intense pulse X-ray deposition, and the evaluation of capability composite materials to resist stress waves, arouse the study of the constitutive relation and the characteristic of stress wave propagating [9]. Reliable numerical simulation results depend on accurate constitutive models as well as effective numerical simulation programs [10]. Among a large number of mechanical numerical simulation programs, the programs used to analyze the material response and structural response related to shock-wave propagation occupy an important position, for example, CTH [11], PUFF-TFT [12], LS-DYNA [13], ABAQUS [14,15], etc. However, since the solid constitutive models of most programs are for isotropic materials, they are not suitable for anisotropic materials. Moreover, the research on the dynamic constitutive model of C/P and its application in numerical simulation is still in the process of update and further perfection. Since the 1990s, many countries have carried out research on the application of anisotropic constitutive models in numerical simulation, and some of the research results have been used in the recent version of the finite element large-scale shock dynamics numerical simulation software, such as LS-DYNA970, that embeds transversely isotropic and orthotropic elastoplastic constitutive models [13]. These models make the software capable of analyzing the dynamic response of fiber-reinforced composites. However, as mentioned above, because of anisotropic mechanical properties, C/P also has a strain rate effect. The interface effect between the fiber and the matrix, damage, temperature, etc., will affect its mechanical properties. Therefore, the establishment of a composite material dynamic constitutive model and improvement of reliability of numerical simulation results are vital to be solved. Lukyanov A et al. [16,17] used the fluid-isotropic ideal elastoplastic constitutive model, and the obtained X-ray plane frontal wave stress peak attenuation curve in the metal is in good agreement with the experiment. However, for C/P, the experimentally measured thermal shock wave stress peak decays faster than numerical simulation. We think this is likely to be the deviation caused by approximating the composite material with anisotropic model in the numerical simulation.

The mechanical properties of C/P composites depend on the properties of their phases and are obviously affected by the manufacturing process. Micromechanics provides a method to calculate the macroscopic mechanical properties of composites based on the properties and geometric shapes of their phases. Therefore, the anisotropy and strain rate correlation is the mechanical properties of most C/P materials, and they will inevitably affect the propagation law of X-ray thermal shock waves. The C/P anisotropic dynamic constitutive model should be introduced in the numerical simulation. In the calculation program. The shell shape of missiles or spacecraft is mostly cylindrical. When exposed to X-ray radiation in the air, the isotropic simplification of composite materials or the one-dimensional simplification of the model will bring errors. Based on the symmetry of the unidirectional X-ray source and the loading shell, it is feasible to adopt a two-dimensional simplified model. However, so far, there is no report about the use of the C/P anisotropic dynamic constitutive model for numerical simulation of two-dimensional X-ray thermal shock wave propagation worldwide. In this study, we used an autonomous program to establish a two-dimensional rate-dependent elastoplastic dynamic constitutive model for orthotropic materials. Taking carbon phenolic (C/P) as an example, the two-dimensional thermal shock waves generated by 1 keV and 3 keV X-ray irradiation were simulated and compared with the measured results. The experimental results show that the anisotropic propagation law of materials is basically consistent with the experimental results.

## 2. Theoretical Basis and Modelling Derivation

### 2.1. Introduction of Numerical Calculation Programme

The thermal shock wave propagation problems are caused by pulsed X-ray irradiation of anisotropic materials. The existing finite element analysis software cannot deal with these problems well. We use the inhouse code “RAMA” to program the two-dimensional finite element dynamics program [8,18] for numerical simulation. The “RAMA” program can deal with material fracture and vaporization problems and embeds a variety of constitutive models and equations of state, including orthotropic dynamic elastoplastic constitutive models. At present, it can simulate the stress wave propagation in the two-dimensional flat plate collision problem of anisotropic and isotropic materials and the two-dimensional X-ray thermal shock wave propagation in various shapes. In the following text, we will discuss in detail the important issues in the program design, such as the spindle rotation of orthotropic materials, the correction of objective stress rate in two-dimensional problems, and the simple algorithm of X-ray energy deposition and so on.

### 2.2. Two-Dimensional Orthotropic Elastoplastic Constitutive Model

Considering the plane strain (taking 1–2 plane strain as an example), there are three main axis directions (expressed by 1, 2, and 3) for orthotropic materials: the fiber warp (warp), the fiber weft (fill), and the thickness direction (thickness) $\varepsilon_{33}=\varepsilon_{13}=\varepsilon_{23}=0,\;\sigma_{13}=\sigma_{23}=0$. The stress–strain relationship is described by Hooke’s law in the elastic denaturation.

(1) $$\left\{ \begin{array}{l} \sigma_{11}=c_{11}\varepsilon_{11}+c_{12}\varepsilon_{22}\\ \sigma_{22}=c_{12}\varepsilon_{11}+c_{22}\varepsilon_{22}\\ \sigma_{33}=c_{13}\varepsilon_{11}+c_{23}\varepsilon_{22}\\ \sigma_{12}=c_{44}\varepsilon_{12} \end{array}\right.$$

$c_{ij}$ is the stiffness matrix coefficient related to the elastic modulus, Poisson ratio, and shear modulus of the material.

There is no corresponding relationship between both strain and stress whose state is related to the deforming path or process in plastic deformation, but the Hooke law is satisfied between stress increment and elastic strain increment. Thus, the constitutive relation is expressed in an incremental form. The strain increment can be decomposed into an elastic strain increment and plastic strain increment, $d\varepsilon_{ij}=d\varepsilon_{ij}^{e}+d\varepsilon_{ij}^{p}$, as shown below:

(2) $$\left[\begin{array}{l} d\sigma_{11}\\ d\sigma_{22}\\ d\sigma_{33}\\ d\sigma_{12} \end{array}\right]=\left[\begin{array}{l} c_{11},c_{12},c_{13},0\\ c_{12},c_{22},c_{23},0\\ c_{13},c_{23},c_{33},0\\ 0,0,0,c_{44} \end{array}\right]\left[\begin{array}{l} d\varepsilon_{11}^{e}\\ d\varepsilon_{22}^{e}\\ d\varepsilon_{33}^{e}\\ d\varepsilon_{12}^{e} \end{array}\right]=\left[\begin{array}{l} c_{11},c_{12},c_{13},0\\ c_{12},c_{22},c_{23},0\\ c_{13},c_{23},c_{33},0\\ 0,0,0,c_{44} \end{array}\right]\left[\begin{array}{l} d\varepsilon_{11}-d\varepsilon_{11}^{p}\\ d\varepsilon_{22}-d\varepsilon_{22}^{p}\\ -d\varepsilon_{33}^{p}\\ d\varepsilon_{12}-d\varepsilon_{12}^{p} \end{array}\right]$$

For isotropic materials, the decoupling of the tolerance and distortion laws by most impingement dynamics programs could be carried out by algorithm, i.e., calculating the static pressure and the partial stress by the state equation and the constitutive relation, respectively. Because of the possible changes in shape and volume by hydrostatic pressure and stress bias, respectively, anisotropic materials cannot be simply decoupled. To reflect the difference from isotropic conditions, the hydrostatic pressure in isotropic materials would be replaced by the mean normal stress $p\left(p=\left(\sigma_{11}+\sigma_{22}+\sigma_{33}\right)/3\right)$ will be used to replace the concept of hydrostatic pressure in isotropic materials. In this paper, stress and strain are defined as positive pressure and negative tension; that is, the average normal stress and deviator stress of orthotropic materials. It can be decoupled in this form to facilitate its application in calculation programs. The stress ($\sigma_{ij}$) is decomposed into mean positive stress (p) and partial stress ($S_{ij}$), as represented by $\;\sigma_{ij}=p\delta_{ij}+S_{ij}$. The strain ($\varepsilon_{ij}$) is decomposed into body strain ($\theta \left(\theta =\varepsilon_{11}+\varepsilon_{22}\right)$) and partial strain ($e_{ij}$), as represented by $\;\varepsilon_{ij}=\frac{1}{3}\theta \delta_{ij}+e_{ij}$. The basic form of the average positive stress at the stage of elastic deformation is shown below:

(3) $$\begin{array}{l} p=\frac{1}{3}(\sigma_{11}+\sigma_{22}+\sigma_{33})\\ p=(c_{11}+3c_{12}+c_{13}+2c_{22}+2c_{23})\frac{\theta }{9}+(c_{11}+c_{13}-c_{22}-c_{23})\frac{e_{11}}{3} \end{array}$$

The basic form of average positive stress increment during plastic deformation is as follows:

(4) $$\begin{array}{c} dp=(c_{11}+3c_{12}+c_{13}+2c_{22}+2c_{23})\frac{d\theta }{9}+(c_{11}+c_{13}-c_{22}-c_{23})\frac{de_{11}}{3}-\\ (c_{11}+c_{12}+c_{13})\frac{d\varepsilon_{11}^{p}}{3}-(c_{12}+c_{22}+c_{23})\frac{d\varepsilon_{22}^{p}}{3}-(c_{13}+c_{23}+c_{33})\frac{d\varepsilon_{33}^{p}}{3} \end{array}$$

In order to make the expression of average normal stress reflect the nonlinear effect of volume change and the anisotropic characteristics of the material, a modified equation of state can be introduced for calculation [19]. Since the X-ray irradiation process not only describes the shock compression state with relatively low temperature but also deals with the state formed by the coupling of X-ray energy deposition and hydrodynamic motion, this work uses the PUFF Equations (5) and (6) of state to describe the specific form [20,21].

Compression zone:

(5) $$\left\{ \begin{array}{l} p=p_{H}+\rho \Gamma (E-E_{H})\\ p_{H}=\frac{\rho_{0}c_{0}^{2}(1-v/v_{0})}{{\left[1-s(1-v/v_{0})\right]}^{2}},E_{H}=\frac{1}{2}p_{H}(v_{0}-v) \end{array}\right.$$

Expansion zone:

(6) $$p=\rho [\gamma -1+(\Gamma_{0}-\gamma +1)\sqrt{\frac{\rho }{\rho_{0}}}][E-E_{S}(1-\exp(N\frac{\rho_{0}}{\rho }(1-\frac{\rho_{0}}{\rho })))]$$

v is the specific volume, ρ is the density, ρ=1/v, Γ is the Grüneisen coefficient, and $c_{0},\;s$ are the fitting experimental parameters in the rational expression of $D=c_{0}+su$ of the material shock wave velocity D and the post-wave particle velocity u. γ is the specific heat ratio, and $E_{s}$ is the sublimation energy, $N=c_{0}^{2}/\Gamma E_{s}$.

Expand Equations (5) and (6) into a polynomial series about $\theta \left(\theta =\varepsilon_{11}+\varepsilon_{22}\approx \left(v_{0}-v\right)/v\right)$ and combine Equation (3) to obtain a correction that can not only reflect the nonlinear characteristics of the material volume change but also reflect the anisotropic strength effect of the material’s equation of state [4]. From this, the corrected average normal stress of the compression zone and expansion zone in the elastic deformation stage is as follows:

(7) $$p=A_{1}^{’}\theta +\left[A_{2}-\frac{\Gamma }{2}A_{1}\right]\theta^{2}+\left[A_{3}-\frac{\Gamma }{2}A_{2}\right]\theta^{3}+(\rho_{0}\Gamma +\rho_{0}\Gamma \theta )E+\frac{1}{3}(c_{11}+c_{13}-c_{22}-c_{23})e_{11}$$

(8) $$p=B_{1}^{’}\theta +B_{2}\theta^{2}+B_{3}\theta^{3}+(\rho_{0}\Gamma +\frac{3}{2}\rho_{0}\Gamma \theta )E-\frac{\gamma -1}{2}\rho_{0}\theta E+\frac{1}{3}(c_{11}+c_{13}-c_{22}-c_{23})e_{11}$$

Therefore, the modified mean normal stress increment of the compression zone and the expansion zone in the plastic deformation stage is obtained by Equation (4).

(9) $$\begin{array}{c} dp=A_{1}^{’}d\theta +2\left[A_{2}-\frac{\Gamma }{2}A_{1}\right]\theta d\theta +3\left[A_{3}-\frac{\Gamma }{2}A_{2}\right]\theta^{2}d\theta +\rho_{0}\Gamma Ed\theta +\\ (\rho_{0}\Gamma +\rho_{0}\Gamma \theta )dE+\frac{1}{3}(c_{11}+c_{13}-c_{22}-c_{23})de_{11}-\frac{1}{3}(c_{11}+c_{12}+c_{13})de_{11}^{p}-\\ \frac{1}{3}(c_{12}+c_{22}+c_{23})de_{22}^{p}-\frac{1}{3}(c_{13}+c_{23}+c_{33})de_{33}^{p} \end{array}$$

(10) $$\begin{array}{ll} dp= & B_{1}^{’}d\theta +2B_{2}\theta d\theta +3B_{3}\theta^{2}d\theta +(\rho_{0}\Gamma +\frac{3}{2}\rho_{0}\Gamma \theta -\frac{\gamma -1}{2}\rho_{0}\theta )dE-\\ & (\frac{3}{2}\rho_{0}\Gamma E-\frac{\gamma -1}{2}\rho_{0}E)d\theta +\frac{1}{3}(c_{11}+c_{13}-c_{22}-c_{23})de_{11}-\\ & \frac{1}{3}(c_{11}+c_{12}+c_{13})de_{11}^{p}-\frac{1}{3}(c_{12}+c_{22}+c_{23})de_{22}^{p}-\frac{1}{3}(c_{13}+c_{23}+c_{33})de_{33}^{p} \end{array}$$

$$\begin{array}{l} A_{1}=\rho_{0}c_{0}^{2},A_{2}=\rho_{0}c_{0}^{2}(2s-1),A_{3}=\rho_{0}c_{0}^{2}(3s^{2}-4s+1),\\ B_{1}=\rho_{0}c_{0}^{2},B_{2}=-\frac{B_{1}}{2}-\frac{\gamma -1}{2\Gamma }B_{1}+\frac{B_{1}}{2}N,\\ B_{2}=\frac{5B_{1}}{24}+\frac{5}{8}\frac{\gamma -1}{\Gamma }-[\frac{5}{4}+\frac{1}{4}\frac{\gamma -1}{\Gamma }]B_{1}N+\frac{1}{6}B_{1}N^{2}\\ A_{1}^{’}=B_{1}^{’}=\frac{1}{9}(c_{11}+3c_{12}+c_{13}+2c_{22}+2c_{33}) \end{array}$$

$A_{1}^{’}\left(B_{1}^{’}\right)$ reflects the material anisotropy or equivalent volume contact and is reduced to volume modulus ($A_{1}\left(B_{1}\right)$) under the isotropic limit condition.

The partial stress ($S_{ij}$) of an elastic stage can be obtained by $S_{ij}=\sigma_{ij}-\left[\left(\sigma_{11}+\sigma_{22}+\sigma_{33}\right)/3\right]\delta_{ij}$ and (1), for example,

(11) $$S_{11}=(2c_{11}+3c_{12}-c_{13}-2c_{22}-2c_{23})\frac{\theta }{9}+(2c_{11}-3c_{12}-c_{13}+c_{22}+c_{23})\frac{e_{11}}{9}$$

The increment of partial stress ($dS_{ij}$) in the plastic stage can be obtained according to Equation (2) and $dS_{ij}=d\sigma_{ij}-\left[\left(d\sigma_{11}+d\sigma_{22}+d\sigma_{33}\right)/3\right]\delta_{ij}$, as shown as follows:

(12) $$\begin{array}{l} dS_{11}=(2c_{11}+3c_{12}-c_{13}-2c_{22}-2c_{23})\frac{d\theta }{9}+(2c_{11}-3c_{12}-c_{13}+c_{22}+c_{23})\frac{de_{11}}{9}-\\ (2c_{11}-c_{12}-c_{13})\frac{de_{11}^{p}}{9}-(2c_{12}-2c_{22}-2c_{23})\frac{de_{22}^{p}}{9}-(2c_{13}-c_{23}-c_{33})\frac{de_{33}^{p}}{9} \end{array}$$

Other expressions of partial stress components can be obtained in the same way. The plastic strain increment of the above formula needs to be solved by yield criterion, and the Tsai–Hill yield criterion can be adopted to determine whether the material has entered the plastic stage for orthogonal anisotropic materials.

(13) $$F=\frac{\sigma_{11}^{2}}{Y_{11}^{2}}+\frac{\sigma_{22}^{2}}{Y_{22}^{2}}+\frac{\sigma_{33}^{2}}{Y_{33}^{2}}+\frac{\sigma_{12}^{2}}{Y_{12}^{2}}+{\bar{Y}}_{33}\sigma_{11}\sigma_{22}+{\bar{Y}}_{22}\sigma_{11}\sigma_{33}+{\bar{Y}}_{11}\sigma_{22}\sigma_{33}-1=0$$

${\bar{Y}}_{11}=\frac{1}{Y_{11}^{2}}-\frac{1}{Y_{22}^{2}}-\frac{1}{Y_{33}^{2}},{\bar{Y}}_{22}=\frac{1}{Y_{22}^{2}}-\frac{1}{Y_{11}^{2}}-\frac{1}{Y_{33}^{2}},{\bar{Y}}_{33}=\frac{1}{Y_{33}^{2}}-\frac{1}{Y_{22}^{2}}-\frac{1}{Y_{11}^{2}},Y_{11},Y_{22},Y_{33},Y_{12}$ are the yield strength in 3 main material directions and the shear yield strength in 1–2 planes.

By the strain rate correlation [17,18], $[\left(F+1\right)/R^{2}]-1=0$ is obtained. $R\left(\varepsilon \right)=1+\beta \ln(\varepsilon /\varepsilon_{0})$ is the strain rate factor, β is the experiment parameters, and $\varepsilon_{0}$ is the reference strain rate. The strain rate (ε) is considered the equivalent plastic strain rate ($\varepsilon_{p}=\sqrt{\frac{2}{3}\varepsilon_{ij}^{p}}$). Thus, the yield criterion of strain rate separation is $f=\varepsilon_{0}\exp[\frac{1}{\beta }\sqrt{F+1}-1)]-\varepsilon_{p}=0$. According to the orthogonal law, it is shown as follows:

(14) $${\dot{\varepsilon }}_{ij}^{p}=\dot{\lambda }\frac{\partial f}{\partial \sigma_{ij}}=\dot{\lambda }\varepsilon_{0}\exp[\frac{1}{\beta }\sqrt{F+1}-1)](\frac{1}{2\beta \sqrt{F+1}})D_{ij}$$

where $D_{ij}=\frac{\partial F}{\partial \sigma_{ij}}$, λ is the plastic flow factor. Based on $\varepsilon_{p}=\sqrt{\frac{2}{3}\varepsilon_{ij}^{p}}=\sqrt{\frac{2}{3}\lambda^{2}\varepsilon_{ij}^{p}}$, we can obtain ($\lambda =\varepsilon_{p}/\sqrt{\frac{2}{3}\frac{\partial f}{\partial \sigma_{ij}}}$). According to the consistency law, the stress state is always on the yield surface when plastic deformation occurs, and we can obtain $\varepsilon_{p}=\varepsilon_{0}\exp[\frac{1}{\beta }\sqrt{F+1}-1)]$ by f=0, as shown below:

(15) $$\lambda =\frac{{\dot{\varepsilon }}_{p}}{\sqrt{\frac{2}{3}\frac{\partial f}{\partial \sigma_{ij}}}}=\frac{2\beta \sqrt{F+1}}{\sqrt{\frac{2}{3}D_{ij}}}\;\mathrm{and}\;D_{ij}^{2}=D_{11}^{2}+D_{22}^{2}+D_{33}^{2}+2D_{12}^{2}$$

The plastic strain rate can be solved based on Equation (14), which is equivalent to the plastic strain increment. Based on this, the average positive stress and partial stress in the elastoplastic stage can be obtained according to corresponding formulas. Such an elastic-plastic constitutive model takes into account not only the rate-related effect but also the nonlinear characteristics of volume change and the anisotropic strength effect of materials in the process of compression and expansion. Thus, it can objectively reflect real material changes.

### 2.3. Energy Deposition of Unit Mass

The interaction between X-rays and matter is essentially the interaction between X-ray photons and matter atoms. The main interaction process is photoelectric effect and Compton scattering, accompanied by fluorescence effect and secondary scattering effect. A parallel X-ray incident vertically along the x-direction onto a 2-D plate should be considered. If $\varphi_{0}$ is the initial energy flux, the spectral energy after the distance x is as follows:

(16) $$\varphi (x)=\varphi_{0}\int_{0}^{\infty }f(\lambda ,T)\exp[-\mu (\lambda )\rho_{x}]d\lambda$$

where $f(\lambda ,T)=\frac{c_{1}}{\lambda^{5}}\frac{1}{\exp(c_{2}/\lambda T)-1}$ is the Plank function. $c_{1}=3.7416\times 10^{-16}\;Wm^{2}$ and $c_{2}=0.014388\;mK$ are the first and second radiation constants. λ is the X-ray wavelength, T is the blackbody spectrum temperature, ρ is the material density, and μ(λ) as the mass absorption coefficient for the material wavelength (λ) depending on the photoelectric effect and Compton scattering.

The continuous energy spectrum is decomposed into several monochromatic lights, and the upper and lower wavelengths should be cut off reasonably for the convenience of numerical calculation. Therefore, Equation (16) can be translated into $\varphi (x)=\varphi_{0}\sum w_{j}\exp[-\mu \left(\lambda \right)\rho -x]$, where $w_{j}$ is the percentage of the total energy spectrum by the group j monochromatic light. So, the energy deposition of unit mass (x(x+Δx),y(y+Δy)) can be calculated by:

(17) $$\Delta E=\frac{[\varphi (x)-\varphi (x+\Delta x)]\Delta y}{\rho \Delta x\Delta y}=\frac{\varphi (x)-\varphi (x+\Delta x)}{\rho \Delta x}$$

**Figure 1:**
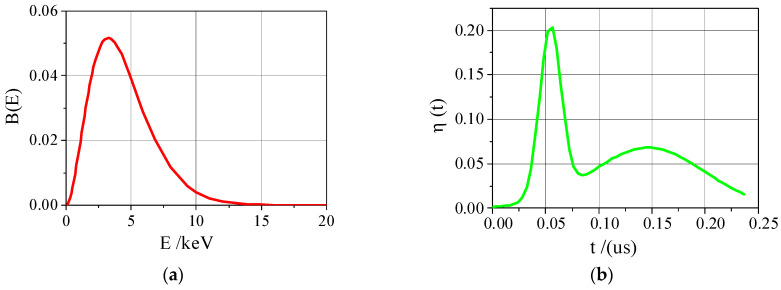
The normalized energy spectrum and time spectrum of intense pulse X-ray. (**a**) energy spectrum; (**b**) time spectrum.

The X-ray energy spectrum and time spectrum are shown in Figure 1. B(E) represents the normalized energy spectrum, and η(t) is the time spectrum, which provides input conditions for subsequent numerical simulation calculations of strong pulse X-rays.

## 3. Experimental Comparison of C/P Materials Irradiated by Low Energy X-rays

**Figure 2:**
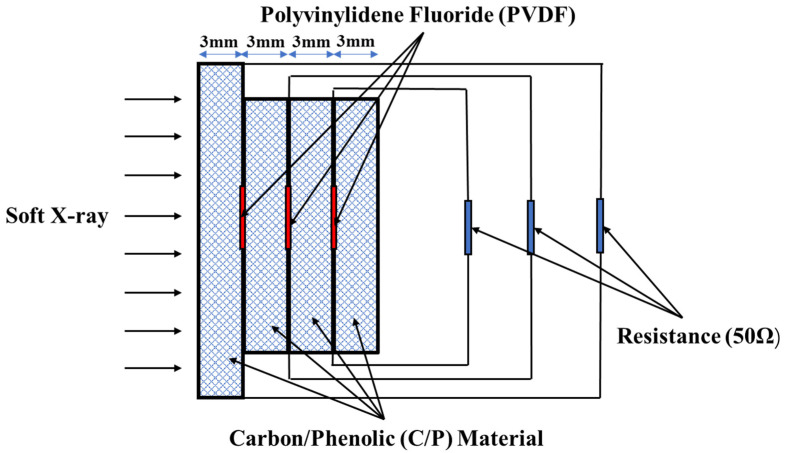
A schematic diagram of PVDF thermal shock wave stress measurement.

In order to verify the two-dimensional orthotropic elastoplastic constitutive model and the energy deposition derivation, a low-energy X-ray irradiation experiment of C/P materials was carried out. The X-ray experimental device is a combined multi-purpose low-energy high-current pulsed electron beam accelerator, which is mainly used to generate pulsed gamma rays and X-rays. According to the energy spectrum and energy fluence output, it can be divided into three X-ray working states. The experiment uses an X-ray mode with an average energy of 0.1–1.5 keV, a pulse width of 40 ns, and a pulse beam energy of 65 KJ. Use Polyvinylidene Fluoride (PVDF) to measure the thermal shock wave of X-rays on carbon phenolic materials. PVDF is a semi-crystalline polymer. The PVDF is directly placed in the target component to directly measure the evolution of the thermal shock wave. As shown in Figure 2, the thickness of the self-made PVDF is about 70 μm, and the influence on the propagation behavior of the thermal shock wave is negligible. PVDF adopts the current mode and connects a load resistance directly to the output end of the membrane, and the signal is transmitted to the oscilloscope to record by the load resistance cable. The oscilloscope adopts high resistance gear, and the shunt of the signal can be ignored. The load R = 50 Ω, the characteristic impedance of the signal cable is 50 Ω, and the two are matched. The relationship between the voltage V(t) recorded by the oscilloscope and the thermal shock wave stress output of PVDF is as follows:

(18) $$\sigma (t)=\frac{1}{\mathrm{AKR}}\int_{0}^{t}V(t)dt$$

where σ(t) is the PVDF thermal shock wave stress output, V(t) is the experimental recording voltage, A is the effective area of the PVDF, K is the dynamic piezoelectric coefficient given by the calibration experiment, and R is the load resistance. At this point, the thermal shock wave waveform can be obtained by integrating the numerical value of the voltage signal recorded by the oscilloscope.

**Figure 3:**
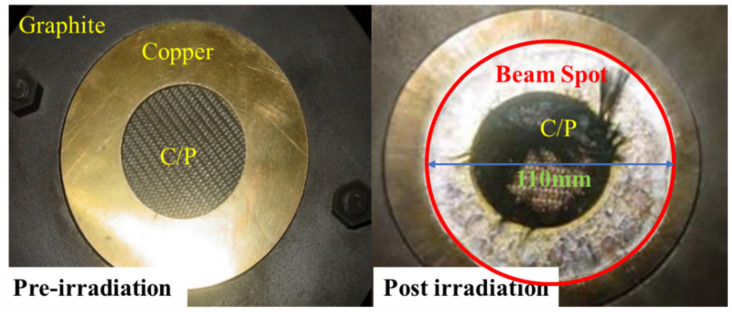
Experimental photos before and after X-ray irradiation.

Figure 3 shows the ablation of the material surface before and after irradiation. After the light-facing surface of the material is irradiated by the electron beam, a large amount of energy is deposited on the surface and converted into heat energy of the substance. The carbon phenolic matrix does not melt after being heated but transforms into a layer of porous carbon. The carbon particles will fly away from the target surface under the action of the sparse wave on the facing surface. Carbon fiber does not melt and only sublimates when its ratio can reach sublimation energy. Therefore, some sublimated carbon fibers will remain on the edge of the target under the ’scouring’ of the jet of carbon particles (as shown in Figure 3). After the experiment, it was found that, from the recovered target, there was spallation on the back side, and some sensors were degummed at the bonding point within the energy fluence range of the experiment. Figure 3 verifies the propagation and effect analysis of thermal shock waves in C/P materials and provides data for the theoretical derivation of numerical calculations.

Table 1 shows the results of five experiments. E is the X-ray average energy, F is the energy fluence, σ is the thermal shock wave stress, and the PVDF installation position is 3 mm, 6 mm, and 9 mm away from the illuminated surface.

**Figure 4:**
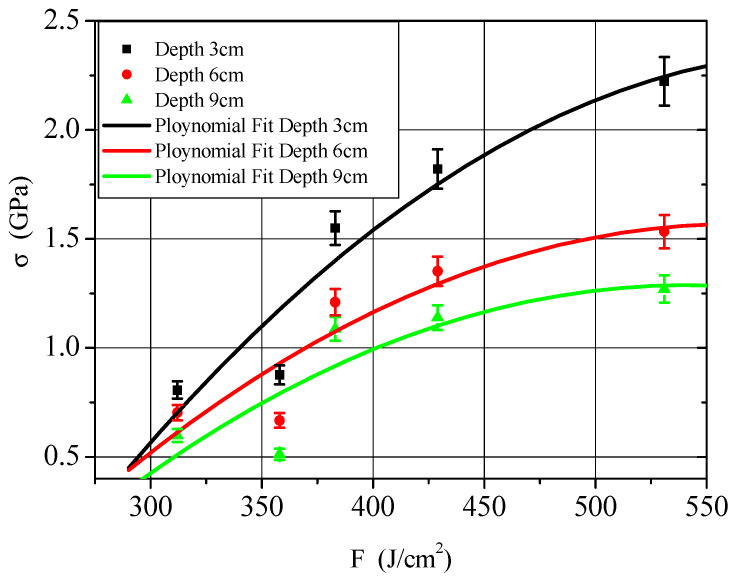
The measured peak stress of thermal shock wave and fitted peak attenuation curve.

Table 1 and Figure 4 show the measured peak stress of the thermal shock wave and the fitted peak attenuation curve and reveal the relationship between the peak stress of the thermal shock wave and the energy fluence at 3, 6, and 9 mm from the target surface in the C/P material. The results show the peak value of the thermal shock wave increases with the increase in energy fluence, but its growth rate not consistent. When the energy fluence is low, the peak stress of the thermal shock wave increases slowly with the increase in the energy fluence, and when the energy fluence is high, its growth accelerates significantly.

**Figure 5:**
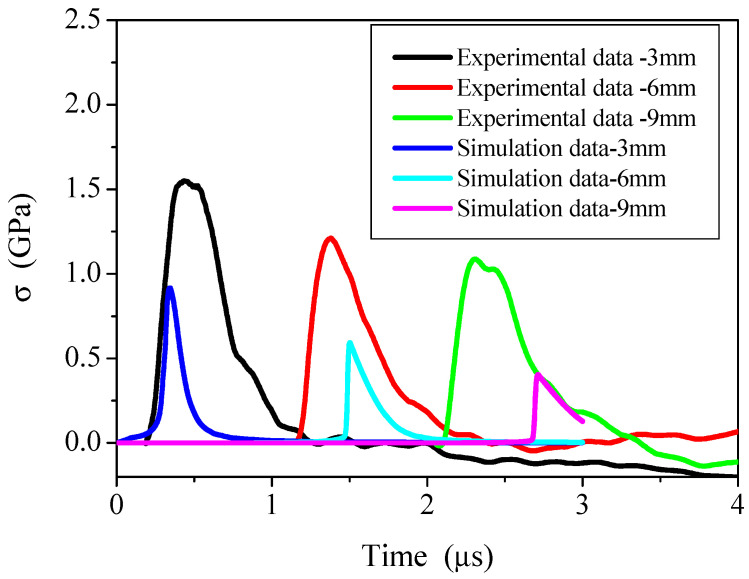
A comparison of C/P material experiment and calculation data.

Figure 5 shows the comparison of thermal shock wave stress experiments and numerical simulation data at 3, 6, and 9 mm in the X direction of C/P materials. The numerical simulation data in Figure 4 uses the calculation results of 1 keV X-ray with similar energy fluence. The experimental energy fluence in the Figure is 383 J/cm^2^, and the numerical simulation energy fluence is 380 J/cm^2^. There is not much difference in between, and the experimental thermal shock wave stress peak value of the same C/P material position is greater than the simulation result. The arrival time of the thermal shock wave coincides with the peak time. When the high current electron accelerator X-ray simulation source is working, the average X-ray energy production is low, which is only 0.4–0.7 keV, while the average energy of the strong pulse 1 keV blackbody spectrum X-ray in the numerical simulation is 2.7 keV. That is because the surface layer of the material has vaporization, the combined effect of vaporization recoil and thermal deformation, the thermal shock wave is mainly manifested as a compression wave. Therefore, the peak pressure of the surface layer is very large, and the stress decays quickly, causing the peak value of the experimental result to be larger than the calculation result. Although the experimental data is limited, it can be determined that the 1 keV blackbody spectrum acts on the surface thermal shock wave stress of the C/P material to be larger (Figure 5). In the experiment, the thermal shock wave stress decay rate is relatively low, so that the experimental thermal shock wave stress is larger than the calculated result when the depth is 3, 6, and 9 mm.

Theoretical analysis shows that under the same energy fluence, the simulated coupling coefficient of X-ray and intense pulsed X-ray in the experiment is greater than 1 keV blackbody spectrum X-ray and intense pulsed X-ray. This is related to the shallower X-ray energy deposition and less vaporized recoil material in the experiment. Therefore, the stress peak value of the thermal shock wave at the deeper position of the X-ray generated by the strong current electron accelerator is greater than the simulation result, which is in line with the law of thermal shock wave generation and propagation.

Through low-energy X-ray experimental research, a qualitatively consistent result with the 1 keV simulation result was obtained, indicating that the calculation method is credible.

## 4. Calculation and Analysis of Thermal Shock Wave for Carbon Phenolic (C/P) Materials

**Figure 6:**
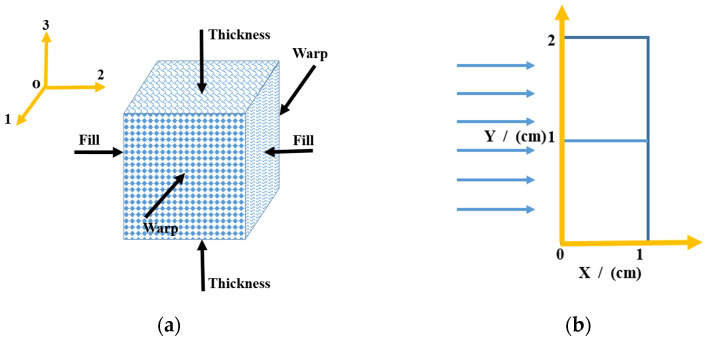
Schematic diagrams of the C/P material model: (**a**) principal directions and lamina of the composite and (**b**) a simplified model for the numerical simulation.

Take carbon phenolic (hereinafter referred to as C/P) plate as an example to simulate the propagation of thermal shock waves under the action of pulsed X-rays. The C/P material is a fiber-reinforced composite material. Its three main axis directions are the warp, fill, and thickness of the fiber cloth. 1, 2, and 3 are used to indicate the material main axis and x, y, and z to represent the system coordinate system. Initially, the two coordinate systems coincide, and the x-direction is taken as the material thickness direction (Thickness), the y-direction is taken as the material warp direction (Warp), and the z-direction is taken as material weft (Fill). As shown in Figure 6a. The size of the C/P board in the x-direction is 1 cm, the size in the y-direction is 2 cm, and the size in the z-direction is much larger than the size in the x and y-directions. X-rays are incident parallel to the x-direction, the irradiation problem can be simplified to a plane strain problem, and the model is shown in Figure 6b.

The parameters of the state equation are shown in Table 2. The main constitutive parameters are shown in Table 3. The mesh size is 0.0025 cm × 0.01 cm. According to the X-ray spectrum, the blackbody spectra of kT = 1 keV and 3 keV are obtained. The initial energy flux is 418 J/cm^2^, and the irradiation time spectrum is a rectangular spectrum with a width of 0.1 μs.

**Figure 7:**
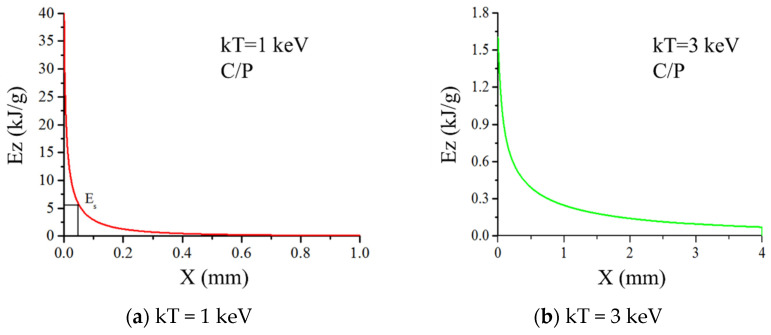
The deposited energy distribution along the symmetry axis under irradiation of X-ray with different blackbody spectra. (**a**) kT = 1 keV; (**b**) kT = 3 keV.

Figure 7 shows the energy deposition profile in carbon phenolic calculated by Equation (17). It can be seen that the energy deposition of 1 keV and 3 keV of X-ray blackbody spectrum decays exponentially, and the peak of energy deposition of soft X-ray of 1 keV is high, the deposition depth is shallow. The 3 keV hard X-ray energy deposition peak is low, but the deposition depth is large.

**Figure 8:**
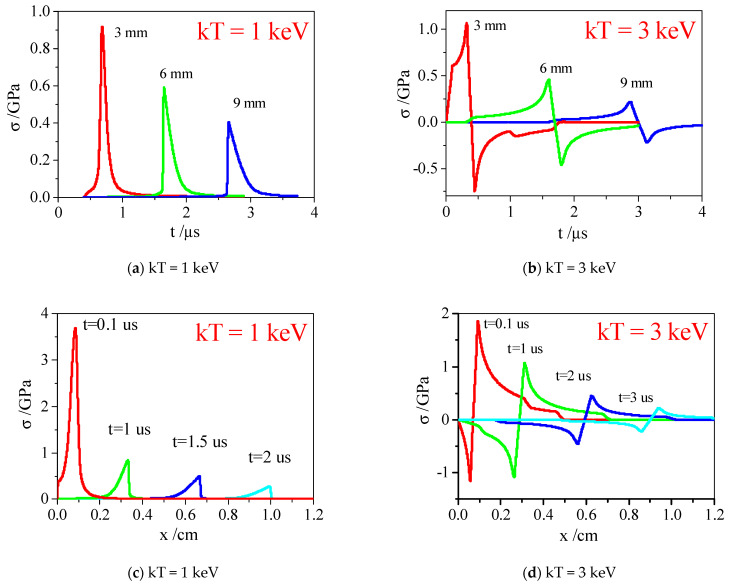
The stress wave along symmetry axis of C/P under radiation of X-ray with different blackbody spectra. (**a**) time distribution, kT = 1 keV; (**b**) time distribution, kT = 3 keV; (**c**) space distribution, kT = 1 keV; (**d**) space distribution, kT = 3 keV.

**Figure 9:**
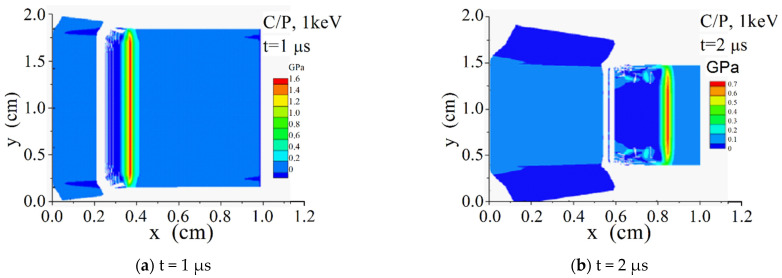
σxx (GPa) contours of C/P under radiation of X-ray with 1 keV blackbody spectrum. (**a**) t = 1 us; (**b**) t = 2 us.

**Figure 10:**
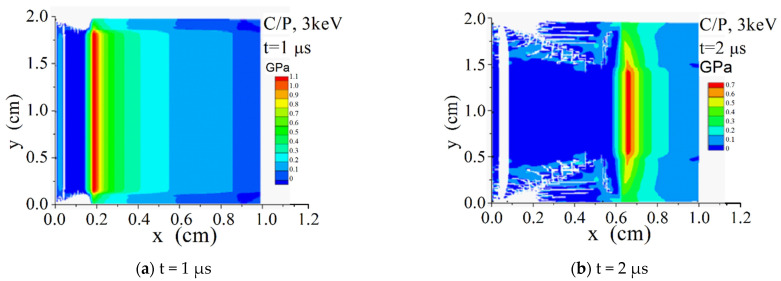
σxx (GPa) contours of C/P under radiation of X-ray with 3 keV blackbody spectrum. (**a**) t = 1 us; (**b**) t = 2 us.

Figure 8 shows the time and space distribution diagrams of σxx along the x-direction on the symmetry axis y = 1 cm in the C/P material, and Figure 9 and Figure 10 show the σxx contour diagrams in the two-dimensional plane.

As shown in Figure 8, the waveform of thermal shock waves is almost triangular, and the full width at half maximum (FWHM) of thermal shock wave expands slightly with wave propagation, with a higher pressure near the material surface and the peak stress value decreasing with wave propagation under different hardness spectrum X-ray irradiation. However, due to the shallow ray penetration under the soft X-ray irradiation of 1 keV, the energy is mainly concentrated near the surface layer. The result shows that the surface layer of the material is vaporized, and the thermal shock wave is mainly expressed as a compression wave due to the combined action of vaporization recoil and thermal deformation. Thus, the peak pressure on the surface is so high, and the attenuation is extremely fast. When the X-ray penetration is deeper, the material would not vaporize under the hard X-ray irradiation of 3 keV. The thermal shock wave is mainly caused by thermal deformation, and the tensile wave is produced after the compression wave. If the X-ray spectrum is hard enough and the flux is large enough, the tensile wave would be much stronger. Therefore, its propagation can cause tensile fracture at any position in the target.

It can be seen from Figure 9 and Figure 10 that the penetration of 3 keV hard X-ray in the material is much deeper than that of 1 keV soft X-ray. The material vaporization takes place under the X-ray irradiation of 1 keV instead of 3 keV, and its tensile strength is much greater. Because it is a 2-D model with an obvious sparse free boundary on its upper and lower sides, the stress wave propagation shows obvious 2-D characteristics.

## 5. Conclusions

In this paper, we use an in-house program containing an orthotropic two-dimensional dynamic elastoplastic constitutive model, take C/P materials as an example, and simulate pulsed X-ray induced thermal shock waves in anisotropic materials. The following conclusions are obtained.

(1) The adopted elastoplastic constitutive model not only considers rate-dependent effects but also considers the nonlinear characteristics of volume changes during compression, expansion, and the anisotropic strength effects of materials, which can objectively reflect the true changes of materials. The theoretical predictions are in good agreement with the experimental results.
(2) Using the X-rays generated by the high current electron accelerator to irradiate the surface of the C/P material, the stress peaks at the thermal shock wave measurement points are larger than the numerical simulation results, which are related to the X-ray energy deposition and stress attenuation rate.
(3) X-ray-induced thermal shock waves in C/P materials have two mechanisms, namely thermal deformation and vaporization recoil. The vaporization recoil phenomenon appears under 1 keV X-ray irradiation, which is mainly manifested as a compression wave. Under 3 keV X-ray irradiation, X-ray penetration is deeper, and the induced thermal shock wave is caused by thermal deformation, resulting in a strong stretching phenomenon, which becomes the main cause of damage to the material.
(4) The thermal shock waveforms in C/P under 1 keV and 3 keV X-ray irradiation, such as the peak value of thermal shock wave, X-ray penetration depth, vaporization phenomenon, tensile strength, etc., are very different. Thermal shock wave propagation exhibits two-dimensional characteristics. The energy deposition of 1 keV and 3 keV both decays exponentially. The energy deposition peak of 1 keV soft X-ray is high, and the deposition depth is shallow; the hard X-ray energy deposition peak of 3 keV is low, and the deposition depth is large.

## Figures and Tables

**Table 1:** The X-ray thermal shock wave experiment results.

NO.	E /keV	F /(J/cm^2^)	σ/GPa		
			3 mm	6 mm	9 mm
1	0.497	383	1.549	1.209	1.087
2	0.422	312	0.807	0.703	0.598
3	0.461	358	0.876	0.667	0.512
4	0.697	531	2.223	1.533	1.270
5	0.587	429	1.821	1.352	1.139

**Table 2:** State parameters of C/P composites.

$\rho_{0}\;(g/{\mathrm{cm}}^{3})$	$c_{0}\;(\mathrm{km}/s)$	s	$\Gamma_{0}$	γ	$e_{s}\;(\mathrm{kJ}/g)$
1.38	2.35	1.66	2.32	1.4	5.15

**Table 3:** Constitutive model parameters of C/P composites.

Elastic Module (GPa)			Shear Module (GPa)	Poisson Ratio			Yield Strength (GPa)				Rate Parameter
$E_{x}$	$E_{y}$	$E_{z}$	$\sigma_{xy}$	$v_{xy}$	$v_{xz}$	$v_{yz}$	$Y_{xx0}$	$Y_{yy0}$	$Y_{zz0}$	$Y_{xy0}$	β
6.96	5.45	4.87	3.5	0.30	0.40	0.313	0.12	0.063	0.17	0.07	0.0218

## Data Availability

All data included in this study are available upon request by contact with the corresponding author.
